# Multimorbidity patterns in the elderly: a prospective cohort study with cluster analysis

**DOI:** 10.1186/s12877-018-0705-7

**Published:** 2018-01-16

**Authors:** Marina Guisado-Clavero, Albert Roso-Llorach, Tomàs López-Jimenez, Mariona Pons-Vigués, Quintí Foguet-Boreu, Miguel Angel Muñoz, Concepción Violán

**Affiliations:** 1 Institut Universitari d’Investigació en Atenció Primària Jordi Gol (IDIAP Jordi Gol), Gran Via Corts Catalanes, 587 àtic, 08007 Barcelona, Spain; 2 Universitat Autònoma de Barcelona, Campus de la UAB, Plaça Cívica, 08193, Bellaterra (Cerdanyola del Vallès), Barcelona, Spain; 3 Gerència d’Àmbit d’Atenció Primària Barcelona Ciutat, Institut Català de la Salut, Carrer de Balmes, 22, Barcelona, Spain; 4 Faculty of Nursing, Universitat de Girona, Emili Grahit, 77, 17071 Girona, Spain; 5 Department of Psychiatry, Hospital Universitari de Vic, Francesc Pla el Vigatà, 1, 08500 Vic, Spain; 6 Unitat de Suport a la Recerca, Institut Universitari d’Investigació en Atenció Primària Jordi Gol (IDIAP Jordi Gol), Carrer de Sardenya, 375, Barcelona, Spain

**Keywords:** Multimorbidity, Chronic disease, Ageing, Primary health care, Cluster analysis, Electronic health record

## Abstract

**Background:**

Multimorbidity is the coexistence of more than two chronic diseases in the same individual; however, there is no consensus about the best definition. In addition, few studies have described the variability of multimorbidity patterns over time. The aim of this study was to identify multimorbidity patterns and their variability over a 6-year period in patients older than 65 years attended in primary health care.

**Methods:**

A cohort study with yearly cross-sectional analysis of electronic health records from 50 primary health care centres in Barcelona. Selected patients had multimorbidity and were 65 years of age or older in 2009. Diagnoses (International Classification of Primary Care, second edition) were extracted using O’Halloran criteria for chronic diseases. Multimorbidity patterns were identified using two steps: 1) multiple correspondence analysis and 2) k-means clustering. Analysis was stratified by sex and age group (65–79 and ≥80 years) at the beginning of the study period.

**Results:**

Analysis of 2009 electronic health records from 190,108 patients with multimorbidity (59.8% women) found a mean age of 71.8 for the 65–79 age group and 84.16 years for those over 80 (Standard Deviation [SD] 4.35 and 3.46, respectively); the median number of chronic diseases was seven (Interquartil range [IQR] 5–10). We obtained 6 clusters of multimorbidity patterns (1 nonspecific and 5 specifics) in each group, being the specific ones: Musculoskeletal, Endocrine-metabolic, Digestive/Digestive-respiratory, Neurological, and Cardiovascular patterns. A minimum of 42.5% of the sample remained in the same pattern at the end of the study, reflecting the stability of these patterns.

**Conclusions:**

This study identified six multimorbidity patterns per each group, one nonnspecific pattern and five of them with a specific pattern related to an organic system. The multimorbidity patterns obtained had similar characteristics throughout the study period. These data are useful to improve clinical management of each specific subgroup of patients showing a particular multimorbidity pattern.

**Electronic supplementary material:**

The online version of this article (10.1186/s12877-018-0705-7) contains supplementary material, which is available to authorized users.

## Background

Multimorbidity is defined as the coexistence of two or more chronic diseases [1, 2]. Although overall life expectancy and healthy life years have increased worldwide, quality of life and functional capacity has worsened [3] due to the chronic conditions strongly related to aging. Some studies predict a rise in prevalence of these conditions [4]; population multimorbidity prevalence currently ranges from 12.9% to 95.1% [5]. In addition, rates of hospitalization and treatment for people with chronic diseases have soared; consequently, a growth in the burden of disease on health systems is assumed in general, and in primary health care in particular [3].

Although life expectancy has increased in the last century [3], research on multimorbidity has been limited and has focused on describing prevalence, estimating severity, and assessing quality of life [6, 7].

In clinical practice, individual patients often present with a collection of chronic diseases which may or may not have a common aetiology, but which require greatly differing and often incompatible management. Prevalence studies, mostly with transversal designs, have identified multimorbidity patterns in patients older than 65 years, but few prospective longitudinal studies have been published and none of them have analysed a period longer than 4 years [5]. With better knowledge about the evolution of multimorbidity patterns, the joint management of several chronic diseases simultaneously could be more effective.

On the other hand, most of the published studies considered diseases, not individuals, as the variable of analysis in assessing multimorbidity patterns. This inhibits an exploration of multimorbidity patterns that takes into account their trajectories and evolution along the individual’s lifetime.

Finally, no consensus has been established about a standard model to determine multimorbidity patterns. Published studies differ in the variables included, such as the unit of analysis selected (patients versus diseases), the statistical method for grouping diseases (factor analysis vs. cluster analysis), diseases included (chronic and/or acute), and number of diseases considered [8, 9]. Nevertheless, non-hierarchical cluster analysis assigns patients into a specified number of clusters [10]. The results are less susceptible to outliers in the data, the influence of the distance measure chosen, or the inclusion of inappropriate or irrelevant variables. Some non-hierarchical cluster analysis methods, like k-means, use algorithms that do not need a distance matrix and can analyse extremely large data sets [10–12].

The aim of this study was to identify multimorbidity patterns over a six-year study period in electronic health records from a Mediterranean urban population older than 65 years and with multimorbidity, attended in primary health care centres in Barcelona (Spain).

## Methods

### Design, setting, and study population

A cohort study with a cross-sectional analysis was carried out in each year of the study period, from 2009 to 2014, in Barcelona, Catalonia (Spain), a city of Mediterranean region with 1,619,337 inhabitants (31/12/2009) [13]. The Spanish National Health Service provides universal coverage, financed mainly by tax revenue. The Catalan Health Institute (CHI) manages 50 primary health care centres (PHCs) in Barcelona that represent 74% of the population [14]. The CHI’s Information System for Research in Primary Care (SIDIAP) contains the clinical information as electronic health records (EHR) recorded by its PHCs since 2006 [15–17].

**Fig. 1:**
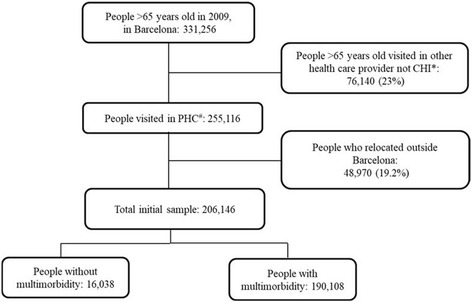
Flow chart of the study

Inclusion criteria were 65–94 years of age on 31 December 2009 and at least one PHC visit during the 6-year study period. From the initial sample of 206,146 (Fig. 1), we excluded people who moved or otherwise sought care outside the CHI system. The only reason to exit the cohort was death (*n* = 24,013), and no new participants were introduced during the study period.

Prevalence of individual conditions varies with age, as does multimorbidity and disease patterns. In order to obtain a more homogenous sample in terms of multimorbidity, we focused on patients from Barcelona city with multimorbidity, defined as 2 or more diagnoses of chronic disease active as of 31 December 2009. We obtained information on that population during 6 years and analysed the data 6 times at cross-sectional time points, every December from 2009 to 2014. However, mortality data were obtained 5 times, from 2010 to 2014.

### Coding and selection of diseases

Diseases are coded in SIDIAP using International Classification of Diseases version 10 (ICD-10). We mapped ICD-10 codes to International Classification of Primary Care, second edition (ICPC-2) codes in order to select chronic diseases by O’Halloran criteria [18] based on the ICPC-2. We only considered chronic diseases with a prevalence over 1% to avoid spurious associations and obtain epidemiologically coherent patterns. Chronic diseases were coded as a dichotomous variable.

### Variables

The unit of measurement was the diagnosis (values: 1 for present, 0 for absent). Other variables recorded for each patient were the following: number of different diseases (chronic diseases active on 31 December each year), age groups in 2009 (65–79; ≥80), and sex (women, men).

### Statistical analysis

Data access: Data were obtained from SIDIAP after the study was authorized. All authors were granted access to the database. No missing values were handled, as sex and age were universally recorded, so there were no missing values and no missing data were imputed. Wrong codes for sex-specific diagnoses and diagnoses with inconsistent dates were excluded.

#### Descriptive analysis

Analyses were stratified by sex and age. Descriptive statistics were used to summarize overall information. Categorical variables were expressed as frequencies (percentage) and continuous as mean (Standard deviation, SD) or median (interquartile range, IQR). Chi-square test and Mann-Whitney test were used to assess differences between age groups by sex.

Prevalence of each chronic disease was calculated for each year in order to study the evolution over time. Multimorbidity patterns were identified using two steps: 1) multiple correspondence analysis (MCA) and 2) k-means clustering. For every year of study (2009–14), MCA and k-means analysis included only those individuals that were alive as of 31 December each year.

#### Multiple correspondence analysis

This data analysis technique for nominal categorical data was used to detect and represent underlying structures in the data set. The MCA method allows representation in a multidimensional space of relationships between a set of dichotomous or categorical variables, in our case diagnoses, that would otherwise be difficult to observe in contingency tables and to show groups of patients with the same characteristics [19, 20]. MCA also allows the direct representation of patients as points (coordinates) in geometric space, transforming the original binary data to continuous data. The MCA analysis was based on the indicator matrix. Optimal number of dimensions extracted and percentages of inertia were determined by scree plot.

#### k-means clustering

From the geometric space created in MCA, patients were classified in clusters according to proximity criteria using the k-means algorithm with random initial centroids. Clusters centres were obtained for each cluster. Optimal number of clusters (k) was assessed according to Calinski Harabaz criteria, using 100 iterations. The optimal number of clusters is the solution with the highest Calinski-Harabaz index value. To assess internal cluster quality, cluster stability of the optimal solution was computed using Jaccard bootstrap values with 100 runs [10]. “Highly stable” clusters should yield average Jaccard similarities of 0.85 and above.

#### Multimorbidity patterns

To describe multimorbidity patterns, frequencies and percentage of diseases in each cluster were calculated. Observed/expected (O/E) ratios were obtained by dividing disease prevalence in the cluster by disease prevalence in each age group, by sex. To define a specific pattern, we considered those diseases with an intra-cluster prevalence ≥20% and an over-expression with O/E ratio ≥ 2 [21]. The names of patterns are related to the main system affected in each cluster.

Descriptive statistics of age and number of diagnoses per each cluster were also obtained. Clinical criteria were used to evaluate the consistency and utility of the final cluster solution, based on clusters previously described in the literature and a consensus opinion drawn from the clinical experience of the research team (3 family physicians and 2 epidemiologists engaged in daily patient care). Stability in the patterns was considered as the number of persons staying in the same pattern in 2014, as well as the percentage of people who remained in the same pattern at the end of the study compared to 2009.

The consistency of multimorbidity patterns was established by analysing the number (percentage) of people who remained stable within the cluster during the study period.

The analyses were carried out using SPSS for Windows, version 18 (SPSS Inc., Chicago, IL, USA) and R version 3.3.1, procedures FactorMineR, fpc, and vegan(R Foundation for Statistical Computing, Vienna, Austria).

## Results

Out of 206,146 persons analysed at the beginning of the study in 2009, 190,108 (92.2%) fulfilled multimorbidity criteria (Fig. 1) and 59.8% were women. The mean age at the beginning of the study was 71.8 (SD 4.35) years for the group 65–79 years old, and 84.2 years (SD 3.46) for the group over 80. In 2009, 31.2% to 39.1% of the population had fewer than 5 chronic diseases, while 40.2% to 42.3% had 6 to 9 diseases and 20.7% to 28.2% had received more than 10 diagnoses. The median number of diseases was 7 (IQR 5–10) for women and for men older than 80 years; the younger men (aged 65–79 years) had a median of 6 diseases (IQR 4–9) (Table 1).

**Table 1:** Number of diseases, stratified by sex and age group

Patients included in the analysis in 2009, *N* = 190,108				
	Women		Men	
	65–79 years	≥80 years	65–79 years	≥80 years
Total, *n*(%)	80,208 (42.2)	33,442 (17.6)	59,331 (31.2)	17,127 (9.0)
Number of diagnoses^*^, *n*(%)				
2	4249 (5.3)	1595 (4.8)	3770 (6.4)	782 (4.6)
[3–5]	22,491 (28.1)	8688 (26.0)	19,429 (32.7)	4534 (26.5)
[6–9]	32,663 (40.7)	13,732 (41.0)	23,846 (40.2)	7252 (42.3)
≥ 10	20,805 (25.9)	9427 (28.2)	12,286 (20.7)	4559 (26.6)
Median number of diagnoses (IQR)^**^	7 (5–10)	7 (5–10)	6 (4–9)	7 (5–10)
Number of diagnoses included in cluster analysis^***^	83	85	84	85
Patients included in the analysis in 2014, *N* = 166,095				
	Women		Men	
	70–79 years	≥80 years	70–79 years	≥80 years
Total *n*(%)	51,835 (31.2)	49,940 (30.1)	38,127 (22.9)	26,193 (15.8)
Number of diagnoses^*^, *n*(%)				
2	3220 (6.2)	2026 (4.1)	2811 (7.4)	1125 (4.3)
[3–5]	16,662 (32.1)	12,144 (24.3)	13,879 (36.4)	6818 (26.0)
[6–9]	21,334 (41.2)	21,316 (42.7)	15,119 (39.6)	11,333 (43.3)
≥ 10	10,619 (20.5)	14,454 (28.9)	6318 (16.6)	6917 (26.4)
Median number of diagnoses (IQR)^**^	6 (4–9)	7 (5–10)	6 (4–8)	7 (5–10)
Number of diagnoses included in cluster analysis^***^	80	84	80	82

Abbreviations: *IQR* Inter-quartile range

^*^Chi-square test; all *p*-values < 0.001

^**^Mann-Whitney test; *P* < 0.001

^***^Chronic diseases with >1% prevalence

### Chronic diseases prevalence

**Fig. 2:**
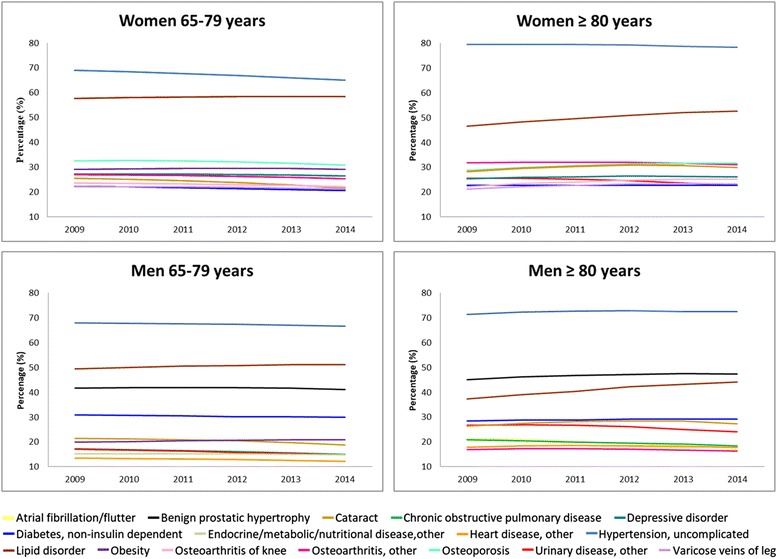
Prevalence of chronic disease across the study period per each age group, stratified by sex

*Hypertension, uncomplicated* was the most prevalent chronic disease in all groups over the period of time studied, followed by *Lipid disorder*. In the group aged 65–79 years, uncomplicated hypertension affected 69% of women and 68% of men in 2009, and lipid disorder affected 57.7% and 49.4%, respectively. Other prevalent diagnoses for women in this age group in 2009 were *Osteoporosis* (32.6%), *Obesity* (29.2%), and *Depressive disorder* (27.3%); among men, ageing-related diseases were prevalent, including *Benign prostatic hypertrophy* (41.6%), *Cataracts* (21.4%), and *Diabetes, non-insulin-dependent (*30.8%). The top 10 chronic diseases for women and men throughout the study period are shown in Fig. 2. Few changes in prevalence were observed over the 6 years analysed.

### K-means clustering

Using the Calinski criterion, six clusters were considered as the optimal solution for both age and sex strata. Average Jaccard bootstrap values for both women and men were 0.85 and above.

### Multimorbidity patterns

For each of the four groups studied (two age groups of men and women), 6 clusters were identified using the k-means method. The first pattern, formed by only the most prevalent diseases, was named the “nonspecific” pattern; the remaining 5 patterns were specific to *Musculoskeletal, Endocrine-metabolic, Digestive/digestive-respiratory, Neuropsychiatric, and Cardiovascular* diseases, in decreasing order depending on the percentage of the population included [see Additional files 1, 2].

The first cluster had the largest percentage of the sample, both women and men: 35.6 and 36.7% of those aged 65–79 years, 34.3–34.1% of those aged 80 and older respectively [see Additional files 1-4]. For women, the top 3 diagnoses throughout the study period were *Hypertension, uncomplicated*; *Lipid disorder;* and *Osteoporosis*. In the older group, *Osteoarthritis, other* was added to the list for the first year and *Cataract* for the other 5 years analysed [see Additional files 1-3].Similarly for men, three diseases predominated in the *Nonspecific* pattern throughout the study period: *Hypertension, uncomplicated*; *Lipid disorder,* and *Benign prostatic hypertrophy*. In older men, these diseases were joined by *Diabetes, non-insulin dependent* in the first year, adding *Cataract* in the remaining 5 years [see Additional files 2, 4]. There was no over-represented disease in these groups (O/E ratio ≥ 2).

Few variations were detected in terms of prevalence and O/E ratios for the elements of a specific cluster, as shown in the example presented in Tables 2 and 3. A pattern observed in women aged 65–79 years was labelled the *Neuropsychiatric* pattern (Table 2). Some neurological diseases were over-represented in 2009, such as *Dementia* (O/E ratio 5.98) or *Stroke/cerebrovascular accident* (O/E ratio 4.81), with a prevalence ≥20%. Other over-represented diseases (O/E ratio ≥ 2) had a prevalence <20% and bear little relation to the main system affected, such as *Ischaemic heart disease without angina* (O/E ratio 4.27, prevalence of 13.9%) or *Atherosclerosis/peripheral vascular disease* (O/E ratio 3.08, prevalence of 9.6%). A large number of patients (in the Table 2, 42.5% of women aged 65–79 years) stayed in the same pattern from baseline until the end of the study period. The rest of these percentages are presented in [see Additional files 1, 2].

**Table 2:** Example of multimorbidity pattern: neuropsychiatric pattern considering observed/expected ratio in one cluster across women aged 65–79 years

Neuropsychiatric pattern		2009		2010		2011		2012		2013		2014	
Number of persons in the cluster		*n* = 8242		*n* = 7493		*n* = 6770		*n* = 6042		*n* = 5360		*n* = 4688	
Persons stable in the pattern. *n*(%)		8242^a^ (100)		7210^a^ (87.5)		6297^a^ (76.4)		5386^a^ (65.4)		4536^a^ (55.0)		3503^a^ (42.5)	
ICPC2	Diagnoses	Prev^b^ (%)	O/E r^c^	Prev^b^ (%)	O/E r^c^	Prev^b^ (%)	O/E r^c^	Prev^b^ (%)	O/E r^c^	Prev^b^ (%)	O/E r^c^	Prev^b^ (%)	O/E r^c^
K86	Hypertension, uncomplicated	70.4	1.02	69.8	1.02	68.7	1.01	68.6	1.03	68.6	1.04	70.8	1.09
T93	Lipid disorder	67.6	1.17	68.4	1.18	68.1	1.17	67.9	1.16	68.4	1.17	70.6	1.21
P76	Depressive disorder	53.5	1.96	53.7	1.97	53.1	1.95	53.4	1.97	51.3	1.90	49.2	1.86
L95	Osteoporosis	39.5	1.21	39.9	1.22	40.0	1.23	39.3	1.22	39.2	1.24	39.2	1.27
P70	Dementia	32.4	*5.98*	30.8	*6.31*	30.1	*6.79*	27.9	*7.05*	25.4	*7.33*	21.0	*6.94*
L91	Osteoarthritis. other	31.8	1.18	31.1	1.16	30.1	1.13	30.4	1.16	29.5	1.14	29.3	1.15
T90	Diabetes. non-insulin dependent	31.0	1.38	30.9	1.40	30.6	1.40	32.8	1.52	33.7	1.60	37.4	1.81
F92	Cataract	29.4	1.15	29.2	1.16	29.1	1.18	29.2	1.22	28.2	1.23	26.4	1.22
K90	Stroke/cerebrovascular accident	23.5	*4.81*	23.1	*4.94*	22.4	*5.02*	21.5	*5.11*	20.2	*5.08*	16.9	*4.50*
P71	Organic psychosis, other	16.0	*5.72*	16.2	*6.06*	16.0	*6.25*	15.8	*6.46*	15.5	*6.61*	14.6	*6.66*
K76	Ischaemic heart disease without angina	13.9	*4.27*	13.9	*4.47*	13.6	*4.57*	14.2	*5.04*	15.4	*5.68*	17.9	*7.00*
K91	Cerebrovascular disease	12.9	*6.05*	12.5	*6.11*	12.4	*6.23*	12.4	*6.33*	12.4	*6.55*	11.5	*6.36*
N87	Parkinsonism	12.8	*6.62*	11.9	*6.51*	12.2	*7.00*	11.6	*7.12*	11.7	*7.47*	11.1	*7.72*
N99	Neurological disease, other	11.6	*4.63*	11.9	*4.85*	11.8	*4.88*	11.8	*5.00*	11.0	*4.78*	10.7	*4.86*
K92	Atherosclerosis/ peripheral vascular disease	9.6	*3.08*	9.6	*3.17*	9.6	*3.30*	9.6	*3.45*	9.4	*3.47*	11.4	*4.36*
K89	Transient cerebral ischaemia	9.3	*3.67*	9.1	*3.74*	8.6	*3.69*	8.7	*3.90*	8.0	*3.77*	6.9	*3.45*
K74	Ischaemic heart disease with angina	8.7	*3.84*	8.9	*3.97*	8.6	*4.03*	9.0	*4.48*	9.1	*4.81*	11.4	*6.54*
K75	Acute myocardial infarction	7.9	*4.79*	8.0	*5.11*	7.7	*5.16*	7.8	*5.60*	8.7	*6.53*	9.6	*7.61*
N88	Epilepsy	6.4	*5.83*	6.5	*6.08*	6.9	*6.45*	6.6	*6.27*	6.4	*6.28*	–	*–*
B81	Anaemia. Vit B12/folate deficiency	4.0	*2.83*	3.7	*2.74*	3.6	*2.78*	3.5	*2.86*	3.5	*3.09*	2.9	*2.80*
F83	Retinopathy	–	–	5.5	*2.12*	5.9	*2.32*	7.2	*2.87*	7.2	*2.96*	9.8	*4.13*
N94	Peripheral neuritis/neuropathy	–	–	–	–	–	–	5.3	*2.15*	5.5	*2.28*	7.0	*2.98*
T82	Obesity	–	–	–	–	–	–	21.1	0.72	21.8	0.74	23.0	0.79
B82	Anaemia. other/unspecified	–	–	–	–	–	–	–	–	13.1	*2.09*	13.9	*2.46*
F84	Macular degeneration	–	–	–	–	–	–	–	–	–	–	3.2	*2.24*

^a^People who stay in the same cluster during the period of study

^b^*Prev*: prevalence

^c^*O/E r*: observed/expected ratio. Italic numbers indicate O/E r ≥ 2

**Table 3:** Example of multimorbidity pattern: neuropsychiatric pattern considering observed/expected ratio in one cluster across men aged 65–79 years

Neuropsychiatric pattern		2009		2010		2011		2012		2013		2014	
Number of persons in the cluster		*n* = 5956		*n* = 5644		*n* = 5080		*n* = 4483		*n* = 3819		*n* = 3274	
Patients stable in the pattern, *n*(%)		5956^a^ (100)		5013^a^ (84.2)		4205^a^ (70.6)		3757^a^ (63.1)		3214^a^ (54.0)		2741^a^ (46.0)	
ICPC2	Diagnosis	Prev^b^ (%)	O/E r^c^	Prev^b^ (%)	O/E r^c^	Prev^b^ (%)	O/E r^c^	Prev^b^ (%)	O/E r^c^	Prev^b^ (%)	O/E r^c^	Prev^b^ (%)	O/E r^c^
K86	Hypertension, uncomplicated	67.3	0.99	70.0	1.03	71.0	1.05	68.9	1.02	68.1	1.02	66.2	0.99
T93	Lipid disorder	54.5	1.10	58.6	1.17	60.6	1.20	59.9	1.18	60.3	1.18	58.7	1.15
Y85	Benign prostatic hypertrophy	45.7	1.10	43.9	1.05	42.4	1.01	42.4	1.02	42.9	1.03	44.5	1.08
T90	Diabetes, non-insulin dependent	37.8	1.23	42.5	1.39	45.5	1.49	43.2	1.43	42.1	1.40	38.2	1.28
K90	Stroke/cerebrovascular accident	34.9	*4.31*	35.4	*4.56*	35.5	*4.73*	35.0	*4.86*	35.3	*5.11*	35.5	*5.34*
P76	Depressive disorder	34.1	*3.11*	32.1	*2.95*	30.4	*2.83*	31.4	*2.94*	31.9	*3.04*	32.3	*3.19*
P70	Dementia	28.2	*6.56*	24.3	*6.24*	22.2	*6.32*	22.2	*6.84*	21.5	*7.52*	21.7	*8.73*
F92	Cataract	23.8	1.11	24.1	1.14	24.3	1.16	23.4	1.14	23.7	1.20	21.2	1.13
K76	Ischaemic heart disease without angina	21.5	*2.12*	24.0	*2.42*	24.4	*2.52*	24.4	*2.57*	22.5	*2.44*	20.0	*2.23*
K92	Atherosclerosis/peripheral vascular disease	19.6	*2.16*	23.4	*2.63*	25.2	*2.92*	23.7	*2.83*	22.6	*2.78*	20.3	*2.60*
K91	Cerebrovascular disease	19.4	*5.84*	20.1	*6.08*	20.9	*6.46*	20.8	*6.57*	21.4	*6.90*	21.2	*7.07*
N87	Parkinsonism	17.3	*6.74*	15.0	*6.14*	13.6	*5.88*	13.6	*6.12*	13.9	*6.66*	14.2	*7.10*
K75	Acute myocardial infarction	13.5	*2.06*	15.8	*2.47*	15.6	*2.50*	15.8	*2.62*	14.9	*2.52*	12.8	*2.28*
N99	Neurological disease, other	13.5	*5.08*	13.0	*4.96*	12.8	*4.98*	13.3	*5.24*	13.9	*5.65*	14.7	*6.22*
P71	Organic psychosis, other	13.5	*6.13*	12.3	*5.77*	11.7	*5.66*	11.4	*5.76*	12.0	*6.35*	12.6	*6.99*
K89	Transient cerebral ischaemia	11.1	*3.58*	10.6	*3.52*	10.3	*3.54*	9.8	*3.50*	9.7	*3.63*	9.0	*3.62*
K74	Ischaemic heart disease with angina	10.3	*2.35*	11.1	*2.58*	11.3	*2.71*	11.9	*2.93*	11.1	*2.79*	9.7	*2.50*
N88	Epilepsy	8.2	*6.13*	7.4	*5.61*	7.1	*5.43*	7.8	*6.06*	8.5	*6.69*	9.1	*7.33*
A04	Weakness/tiredness. General	6.4	*2.63*	6.1	*2.50*	6.0	*2.53*	5.7	*2.51*	5.9	*2.83*	5.7	*3.13*
B81	Anaemia. Vit B12/folate deficiency	4.0	*2.63*	3.9	*2.65*	3.9	*2.79*	3.1	*2.39*	2.8	*2.20*	2.6	*2.12*
B82	Anaemia. other/unspecified	–	–	14.5	*2.01*	14.8	*2.13*	13.7	*2.08*	–	–	–	–
F83	Retinopathy	–	–	6.5	*2.13*	7.4	*2.45*	6.6	*2.17*	6.4	*2.09*	–	–
N94	Peripheral neuritis/neuropathy	–	–	6.2	*2.07*	6.5	*2.22*	6.6	*2.25*	6.5	*2.28*	5.7	*2.07*
U99	Urinary disease. Other	–	–	20.4	1.22	20.3	1.24	–	–	–	–	–	–

^a^People who stayed in the same cluster throughout the period of study

^b^*Prev*: prevalence

^c^*O/E r*: observed/expected ratio. Italic numbers indicate O/E r ≥ 2

Table 3 shows men aged 65–79 years with the *Neuropsychiatric* pattern, containing almost the same diseases as the homologous pattern in women. Differences between the patterns are mainly sex-related diseases such as *Benign prostatic hypertrophy*.

Following the same method as these two examples, it can be observed that chronic diseases included in each pattern at the beginning of the sample mostly persisted throughout the 6 years analysed. Some variations were observed, such as chronic disease leaving the pattern when it did not meet the inclusion criteria, sometimes only by a few decimal points that decided whether a disease remained in a pattern or not [see Additional files 1-4].

Among women aged 80 and older, as in the younger group, we defined six clusters (*Nonspecific* and 5 specific multimorbidity patterns) with the same names, even if the diseases varied, because the main system affected was the same. The *Muskuloskeletal, Endocrine-metabolic, Digestive and Cardiovascular* patterns showed changes in 1 or 2 diseases, but the *Neuropsychiatric* pattern had added 4 diseases to the cluster by the end of the study period [see Additional file 3].

Several differences were observed in the older group of men, as well. First, the *Endocrine-metabolic* pattern in this age group was defined by diseases localized in the *Cardiovascular* patterns in men aged 65–79 years. Secondly, the *Digestive* pattern incorporated respiratory diseases, becoming the *Digestive-respiratory* pattern (as in the last year analysed in men 65–79 years), composed of 9 more chronic diseases than the *Digestive* pattern. Thirdly, the *Neuropsychiatric* and *Cardiovascular* patterns lost some diseases. Finally, no important changes were found in the *Musculoskeletal* pattern [see Additional file 4].

**Fig. 3:**
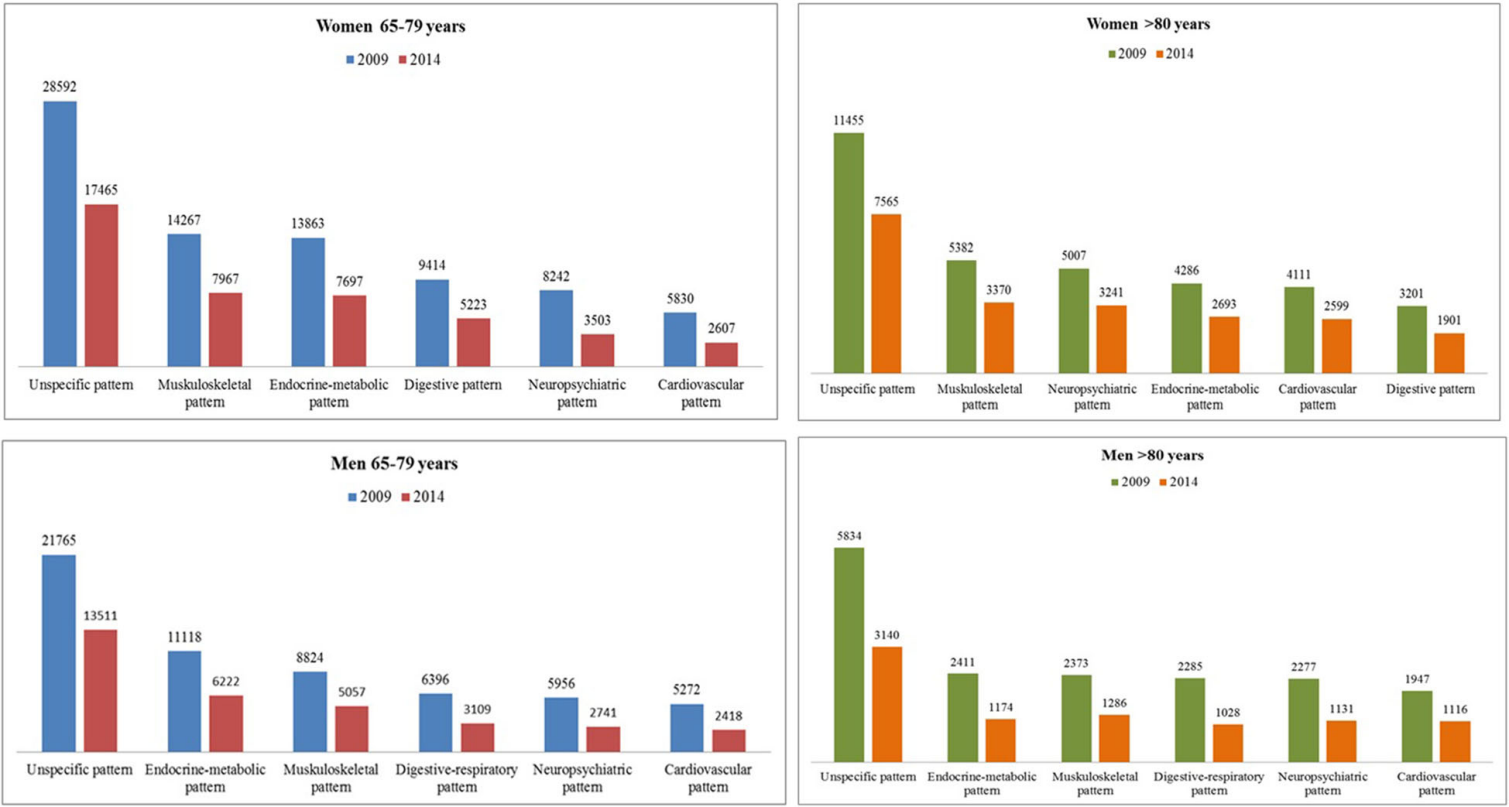
Sample corresponding to each pattern and people remaining in that pattern at the end of the study

Furthermore, the percentage of patients whose multimorbidity pattern remained stable exceeded 42.5% for all patterns per each sex and age group. The *Nonspecific* patterns had the highest values for stability at the end of the period for all groups except men aged 80 and older, for which the cardiovascular pattern was the highest (Fig. 3).

## Discussion

We explored multimorbidity patterns and their 6-year evolution in people aged 65 years and older with multimorbidity attended in PHC. The most prevalent chronic diseases, *Hypertension, uncomplicated* and *Lipid disorder*, were represented in all clusters in all four groups (i.e., men and women aged 65–79 and ≥80 years). We found 6 clusters per group, 5 of them with a specific pattern related to an organic system: *Musculoskeletal, Endocrine-metabolic, Digestive/Digestive-respiratory, Neuropsychiatric* and *Cardiovascular* patterns. We analysed multimorbidity patterns over 6 years and found that they remained quite similar from the beginning to the end of the study period.

We observed a high prevalence of multimorbidity in our population sample, with a higher proportion for women, as in other published studies [5, 8] and described 6 patterns in each study group. In addition, the prevalence of chronic diseases and multimorbidity patterns was similar to previous studies in Catalonia [22] and in other developed countries [23–25]. In a separate study in the same sample, we analysed mortality rates and observed higher mortality among men with *Digestive-respiratory* patterns and among women with *Cardiovascular* pattern [26].

In both age groups, both men and women had the same 5 multimorbidity pattern names plus one additional cluster: a *Digestive* disease pattern in women and a *Digestive-respiratory* pattern in men. This difference is probably related to the smoking and alcohol habits that were more common among men than among women in the age groups studied [27]. The differences observed between age groups were related to disease prevalence and O/E ratio; no significant differences between men and women were found in the systems that were most commonly affected by the prevalent diseases. As a result, future clinical guidelines could focus on improving common management of multimorbidity in all older patients.

It is particularly noteworthy that more than 50% of those showing the *Nonspecific* pattern remained in that same pattern across the period analysed, without moving on to a specific pattern; a few degenerative diseases were added in the older groups. In addition, this first (*Nonspecific*) cluster was defined by highly prevalent diseases, with no over-represented chronic diseases, so that the association between diseases could exist by chance. Consequently, this first cluster showed that a considerable portion of the sample had no system-specific pattern.

In contrast, across the specific patterns we also observed a large proportion (range from 42.5 to 64.7%) of people remaining stable (in terms of chronic disease prevalence) in the same pattern. Maximum stability was observed for the *Nonspecific* pattern in both groups aged 65 to 79 years and in older women; for men aged 80 and older, the *Cardiovascular* pattern showed the greatest stability. Moreover, some people changed from one pattern to another but the multimorbidity pattern kept mostly stable during the 6 years studied, confirming the long-term stability of the multimorbidity pattern composition. In view of these results, an association could be hypothesized between multimorbidity and specific genetic conditions, as well as previously suggested associations with lifestyle and environmental conditions [28].

Estimates of multimorbidity pattern prevalences differ deeply in the literature because of variations in methods, data sources and structures, populations and diseases studied. Although this makes it challenging to compare study results [5, 29, 30], there are some similarities between the present and previous studies. For instance, the most common organic systems affected in previous studies of multimorbidity patterns were cardiovascular/metabolic, neuropsychiatric (mental health), and musculoskeletal [30]. Our study found patterns affecting these same organic systems; however, it offers another point of view for defining multimorbidity patterns. Cluster analysis shows the complexity of multimorbidity in persons aged 65 years and older and is likely to be helpful in shaping future strategies to continue studying this important health issue.

Previous studies have analysed no more than four years of data [29], compared to six years of information about the evolution of a multimorbidity pattern in our study. As a result, we identified long-term stability in multimorbidity patterns, observing some differences between age groups, related to prevalence and O/E ratio in chronic diseases. Useful information can be extracted from our study for the monitoring and treatment of each multimorbidity pattern.

### Strengths and limitations

A major strength of this study is the analysis of a large, high-quality EHR database, representative of a large population. In the context of a national health system with universal coverage, EHR data have been shown to yield more reliable and representative conclusions than those derived from survey-based studies [25]. The inclusion of all chronic diagnoses registered in EHR contributed to a more accurate analysis of the multimorbidity patterns in this population. Moreover, the use of data collected by the primary health care system increased the external validation of the information extracted because primary care centres in Barcelona attended more than 70% of the population at least once a year during the study period. As the nonspecific pattern contained well-known chronic diseases with established clinical guidance, the information extracted is relevant but less useful in clinical practice than the specific patterns defined. The long time period observed provided information on the stability of the patterns during six years, enabling us to focus on creating better strategies to address all five specific patterns in terms of prevention, diagnosis, and treatment of these systemic clusters of prevalent diseases.

A number of limitations must be taken into account as well. First, EHR accuracy depends on the data entered by each general physician or nurse, and EHR systems are not designed as general-purpose research tools [31]. Another weakness could be the attention only to chronic diseases, which precludes awareness of acute diseases or bio-psychosocial factors [2]. Nonetheless, the inclusion of a wide range of diseases makes it possible to find multimorbidity patterns not previously obtained and increases complexity in terms of assembling patterns. Finally, we did not have data on cause of death.

In addition, using MCA can produce low percentages of variation on principal axes, complicating the choice of the number of dimensions to retain. We assumed a five-dimension solution, using the elbow rule in the scree plot to have the most accurate solution possible without including an unwieldy number of dimensions in the analysis [19]. Although we did not retain the total variance of the dataset, clustering techniques can be applied to the reduced dataset while preserving its complexity.

The strength of using k-means cluster analysis is that the results are less susceptible to outliers in the data, the influence of the chosen distance measure, or the inclusion of inappropriate or irrelevant variables. The method can also analyse extremely large data sets (as in this study), as no distance matrix is required. On the other hand, some disadvantages of the method are that different solutions can occur for each set of seed points and there is no guarantee of optimal clustering [11]. To minimize this shortcoming, we tested the internal validity of our solution using bootstrap methods [32], and the results were highly stable (Jaccard > 0.85). However, the method is not efficient when a large number of potential cluster solutions are to be considered [11]; to address this limitation, we computed the optimal number using analytical indexes like Calinski Harabasz [33].

### Future research

With this confirmation of the stability of multimorbidity patterns across age groups, sex, and time, some actions could be considered to improve multimorbidity management. For instance, clinical guidance could encompass a specific pattern to deal with its complexity rather than creating multiple guidelines for each of the chronic diseases. Relevant information could be extracted from our study for the monitoring and treatment of each multimorbidity pattern. Finally, genetic factors, as well as socioeconomic status, should be taken into account in future studies.

## Conclusions

We identified a very large proportion of people over 65 years with multimorbidity, distributed in six clusters; five affected a specific system in the body and one had a nonspecific pattern. The major portion of the sample fit this last pattern, which had few diseases; this finding could be related to genetic or social characteristics of the sample. On the other hand, stability in a specific pattern over an extended time period might give us the information needed to take a new approach and improve a patient’s situation. For instance, a new clinical practice guideline could be developed to control a combination of chronic diseases rather than each one individually.

As the prevalence of chronic diseases was stable over the period studied, multimorbidity patterns also became firmer. Therefore, the k-means technique is useful to analyse multimorbidity patterns in real-world data.

The observation that multimorbidity patterns are constant over time is very useful for the specific clinical management of each patient who fits a specific multimorbidity pattern. Further studies using this method in other groups of patients should be performed to validate the results obtained.

## Additional files

Additional file 1: Multimorbidity patterns in women 65–79 years across the period analysed. Patterns defined by prevalence >20% and ratio O/E > 2. (XLSX 26 kb)

Additional file 2: Multimorbidity patterns in men 65–79 years across the period analysed. Patterns defined by prevalence >20% and ratio O/E > 2. (XLSX 32 kb)

Additional file 3: Multimorbidity patterns in women aged >80 years across the period analysed. Patterns defined by prevalence >20% and ratio O/E > 2. (XLSX 29 kb)

Additional file 4: Multimorbidity patterns in men >80 years across the period analysed. Patterns defined by prevalence >20% and ratio O/E > 2. (XLSX 37 kb)

## Abbreviations

CHI: Catalan Health Institute
EHR: Electronic health records
ICD-10: International Classification of Diseases version 10
ICPC-2: International Classification of Primary Care second edition
IDIAP Jordi Gol: Institut Universitari d’Investigació en Atenció Primària Jordi Gol
IQR: Interquartile range
MCA: Multiple Correspondence Analysis
O/E ratios: Observed/Expected ratios
PHCs: Primary health care centres
SD: Standard deviation
SIDIAP: Information System for Research in Primary Care

## References

[1] Valderas JM, Sibbald B, Salisbury C (2009). Defi ning Comorbidity: implications for understanding health and health services. Ann Fam Med.

[2] Le Reste JY, Nabbe P, Rivet C, Lygidakis C, Doerr C, Czachowski S (2015). The European general practice research network presents the translations of its comprehensive definition of multimorbidity in family medicine in ten European languages. PLoS One.

[3] Murray CJL, Barber RM, Foreman KJ, Ozgoren AA, Abd-Allah F, Abera SF (2015). Global, regional, and national disability-adjusted life years (DALYs) for 306 diseases and injuries and healthy life expectancy (HALE) for 188 countries, 1990–2013: quantifying the epidemiological transition. Lancet.

[4] Aboderin I, Kalache A, Ben-Shlomo Y, Lynch JW, Yajnik CS, Kuh D, et al. (2002) Life course perspectives on coronary heart disease, stroke and diabetes: key issues and implications for policy and research. Geneva, World Health Organization. http://apps.who.int/iris/bitstream/10665/67174/1/WHO_NMH_NPH_02.1.pdf Accessed 19 July 2017.

[5] Violan C, Foguet-Boreu Q, Flores-Mateo G, Salisbury C, Blom J, Freitag M (2014). Prevalence, determinants and patterns of multimorbidity in primary care: a systematic review of observational studies. PLoS One.

[6] Bayliss EA, Ellis JL, Steiner JF (2005). Subjective assessments of comorbidity correlate with quality of life health outcomes: initial validation of a comorbidity assessment instrument. Health Qual Life Outcomes.

[7] Fortin M, Bravo G, Hudon C, Lapointe L, Almirall J, Dubois MF (2006). Relationship between multimorbidity and health-related quality of life of patients in primary care. Qual Life Res.

[8] Marengoni A, Angleman S, Melis R, Mangialasche F, Karp A, Garmen A (2011). Aging with multimorbidity: a systematic review of the literature. Ageing Res Rev.

[9] Holzer BM, Siebenhuener K, Bopp M, Minder CE (2017). Evidence-based design recommendations for prevalence studies on multimorbidity: improving comparability of estimates. Popul Health Metr.

[10] Everitt BS, Landau S, Leese M, Stahl D. Cluster analysis. 5th ed. Chichester: Wiley; 2011; p. 321–30.

[11] Liao M, Li Y, Kianifard F, Obi E, Arcona S (2016). Cluster analysis and its application to healthcare claims data: a study of end-stage renal disease patients who initiated hemodialysis. BMC Nephrol.

[12] Ilmarinen P, Tuomisto LE, Niemelä O, Tommola M, Haanpää J, Kankaanranta H (2017). Cluster analysis on longitudinal data of patients with adult-onset asthma. J Allergy Clin Immunol Pract.

[13] Official numbers of population in Barcelona. http://www.bcn.cat/estadistica/castella/dades/tpob/pad/ine/a2010/edat/edatq01.htm Accessed 19 July 2017.

[14] Memòria 2013. Institut Català de la Salut. http://ics.gencat.cat/web/.content/documents/memoriesICS/ics2013.pdf Accessed 20 Jul 2017.

[15] Del Mar G-GM, Hermosilla E, Prieto-Alhambra D, Fina F, Rosell M, Ramos R (2012). Construction and validation of a scoring system for the selection of high-quality data in a Spanish population primary care database (SIDIAP). Inform Prim Care.

[16] Prieto-Alhambra D, Judge A, Javaid MK, Cooper C, Diez-Perez A, Arden NK (2014). Incidence and risk factors for clinically diagnosed knee, hip and hand osteoarthritis: influences of age, gender and osteoarthritis affecting other joints. Ann Rheum Dis.

[17] Ramos R, Balló E, Marrugat J, Elosua R, Sala J, Grau M (2012). Validity for use in research on vascular diseases of the SIDIAP (information system for the development of research in primary care): the EMMA study. Rev Esp Cardiol (Engl Ed).

[18] O’Halloran J, Miller GC, Britt H (2004). Defining chronic conditions for primary care with ICPC-2. Fam Pract.

[19] Sourial N, Wolfson C, Zhu B, Quail J, Fletcher J, Karunananthan S (2010). Correspondence analysis is a useful tool to uncover the relationships among categorical variables. J Clin Epidemiol.

[20] García-Gil M, Blanch J, Comas-Cufí M, Daunis-i-Estadella J, Bolíbar B, Martí R (2016). Patterns of statin use and cholesterol goal attainment in a high-risk cardiovascular population: a retrospective study of primary care electronic medical records. J Clin Lipidol.

[21] Schäfer I, Kaduszkiewicz H, Wagner H-O, Schön G, Scherer M, van den Bussche H (2014). Reducing complexity: a visualisation of multimorbidity by combining disease clusters and triads. BMC Public Health.

[22] Foguet-Boreu Q, Violán C, Rodriguez-Blanco T, Roso-Llorach A, Pons-Vigués M, Pujol-Ribera E (2015). Multimorbidity patterns in elderly primary health care patients in a South Mediterranean European region: a cluster analysis. PLoS One.

[23] Britt HC, Harrison CM, Miller GC, Knox SA. Prevalence and patterns of multimorbidity in Australia. Med J Aust. 2008;189(2):72–7. 10.5694/j.1326-5377.2008.tb01919.x 18637770

[24] Barnett K, Mercer SW, Norbury M, Watt G, Wyke S, Guthrie B (2012). Epidemiology of multimorbidity and implications for health care, research, and medical education: a cross-sectional study. Lancet.

[25] Violán C, Foguet-Boreu Q, Hermosilla-Pérez E, Valderas JM, Bolíbar B, Fàbregas-Escurriola M (2013). Comparison of the information provided by electronic health records data and a population health survey to estimate prevalence of selected health conditions and multimorbidity. BMC Public Health.

[26] Ibarra-Castillo C, Guisado-Clavero M, Violan Fors C, Pons-Vigués M, López-Jiménez T, Roso-Llorach A. Survival in relation to multimorbidity patterns in older adults in primary care in Barcelona, Spain (2010-2014): A longitudinal study based on electronic health records. J Epidemiol Community Health. (in press). 10.1136/jech-2017-209984 29330165

[27] Lopez AD, Collishaw NE, Piha T (1994). A descriptive model of the cigarette epidemic in developed countries. Tob Control.

[28] Hu JX, Thomas CE, Brunak S (2016). Network biology concepts in complex disease comorbidities. Nat Rev Genet.

[29] France EF, Wyke S, Gunn JM, Mair FS, McLean G, Mercer SW (2012). Multimorbidity in primary care: a systematic review of prospective cohort studies. Br J Gen Pract.

[30] Prados-Torres A, Calderón-Larrañaga A, Hancco-Saavedra J, Poblador-Plou B, Van Den Akker M (2014). Multimorbidity patterns: a systematic review. J Clin Epidemiol.

[31] Coorevits P, Sundgren M, Klein GO, Bahr A, Claerhout B, Daniel C (2013). Electronic health records: new opportunities for clinical research. J Intern Med.

[32] Hennig C (2007). Cluster-wise assessment of cluster stability. Comput Stat Data Anal.

[33] Caliński T, Harabasz J (2007). A dendrite method for cluster analysis. Commun Stat Methods.

